# Genome-wide differences in DNA methylation changes in caprine ovaries between oestrous and dioestrous phases

**DOI:** 10.1186/s40104-018-0301-x

**Published:** 2018-12-04

**Authors:** Xiaopeng An, Haidong Ma, Peng Han, Chao Zhu, Binyun Cao, Yueyu Bai

**Affiliations:** 1 College of Animal Science and Technology, Northwest A&F University, No. 22 Xinong Road, Yangling, Shaanxi 712100 People’s Republic of China; 2 Animal Health Supervision Institute of Henan Province, No. 91 Jingsan Road, Zhengzhou, Henan 450008 People’s Republic of China

**Keywords:** Differentially methylated genes, Gene ontology, Oestrous cycle, Pathway

## Abstract

**Background:**

DNA methylation plays a vital role in reproduction. Entire genome DNA methylation changes during the oestrous phase (ES) and dioestrous phase (DS) in the ovaries of Guanzhong dairy goats were investigated using bisulphite sequencing to understand the molecular biological mechanisms of these goats’ oestrous cycle.

**Results:**

We discovered distinct genome-wide DNA methylation patterns in ES and DS ovaries. A total of 26,910 differentially methylated regions were upregulated and 21,453 differentially methylated regions were downregulated in the ES samples compared with the DS samples (*P*-values ≤0.05 and fold change of methylation ratios ≥2). Differentially methylated region analysis showed hypomethylation in the gene body regions and hypermethylation in the joining region between upstream regions and gene bodies. The methylation ratios of the *STAR*, *FGF2*, *FGF12*, *BMP5* and *SMAD6* genes in the ES samples were lower than those of the DS samples (*P*-values ≤0.05 and fold change of methylation ratios ≥2). Conversely, the methylation ratios of the *EGFR*, *TGFBR2*, *IGF2BP1* and *MMD2* genes increased in the ES samples compared with the DS samples. In addition, 223 differentially methylated genes were found in the GnRH signalling pathway (KO04912), ovarian steroidogenesis pathway (KO04913), oestrogen signalling pathway (KO04915), oxytocin signalling pathway (KO04921), insulin secretion pathway (KO04911) and MAPK signalling pathway (KO04010).

**Conclusions:**

This study is the first large-scale comparison of the high-resolution DNA methylation landscapes of oestrous and dioestrous ovaries from dairy goats. Previous studies and our investigations have shown that the *NR5A2*, *STAR*, *FGF2* and *BMP5* genes might have potential application value in regulating caprine oestrus.

**Electronic supplementary material:**

The online version of this article (10.1186/s40104-018-0301-x) contains supplementary material, which is available to authorized users.

## Background

DNA methylation is an important epigenetic mechanism involved in diverse biological processes, including cell differentiation [1], tumourigenesis [2, 3], gametogenesis and early embryogenesis [4, 5], X chromosome inactivation [6, 7] and cell apoptosis [8]. Methylation patterns vary across different breeds and sexes of domestic animals, as well as in different anatomical tissues, and can manifest as phenotypic changes [9, 10]. Addition of a methyl group to the fifth carbon of a cytosine base (5mC) and adenine methylation are the most common forms of DNA methylation. The most studied form is CpG methylation, while other forms of cytosine methylation include CHH and CHG (where C = Cytosine; H = Adenine, Guanine, or Thymine; and G = Guanine) [11]. Approximately 60% of genes in the mammalian genome show high CpG density in their promoters. DNA methylation in promoters inhibits gene expression by reducing the rate of transcriptional elongation [12, 13]. In general, methylation in the immediate vicinity of the transcription start site (TSS) blocks initiation of transcription, but this phenomenon is dependent on the binding transcription factors [13, 14]. Methylation in the gene body influences gene expression by stimulating transcription elongation and alternative splicing [15, 16]. Studying the distribution of DNA methylation in the genome is a prerequisite for understanding the function of epigenetics. Previous studies have focused on genome-wide DNA methylation to explore tissue-specific methylation associated with economically important traits in livestock, for example experiments have investigated the muscle tissue of sheep and cattle [17, 18], placentas of cows [19], adipose and muscle tissue of pigs [20], porcine ovaries [10], and milk fat production in dairy goats [21].

The caprine oestrous cycle lasts approximately 21 days and includes four distinct stages, namely dioestrous, pro-oestrous, oestrous and meta-oestrous [22]. The hypothalamic-pituitary-gonadal axis plays a key role in regulating the oestrous cycle [23]. Dynamic changes in DNA methylation occur in the reproductive organs and during regulated sexual and ovarian maturation [24, 25]. The proximal promoter of the aromatase *CYP19* gene can be hypomethylated during follicular development but was found to be hypermethylated during luteinisation in buffalo ovaries [26]. Other previous work has shown that during puberty DNA methylation declines in the hypothalamus of goats [27]. The ovary has gained increasing research attention because it is responsible for egg maturation and other changes in biological structures and their morphology during the oestrous cycle. The main processes in the ovary during dioestrous are corpus luteum growth, egg maturation, and ovulation during oestrous, under the stimulation of gonadotropins [22, 28, 29]. A few genes crucial for ovarian activity exhibit different methylation levels during the oestrous cycle; these genes include *FOXL12* for folliculogenesis [30], *PGF2α* for luteolysis [31] and *EREG* for oocyte maturation [32]. Hypermethylation of CCGG and GATCG sites has been noted in most granulosa cells of tertiary follicles compared with those of primary and secondary follicles [33]. A total of 90 differentially methylated genes were significantly enriched in biological processes and pathways related to reproduction and hormone regulation, with differentially methylated genes associated with regulating reproduction in pigs [34]. Although studies have confirmed that DNA methylation plays a key role in ovarian activity, changes in genome-wide DNA methylation in the ovaries of dairy goats during their oestrous cycle have not been investigated. In the present study, we investigated genome-wide DNA methylation profiles in caprine ovaries during the oestrous (ES) and dioestrous phases (DS), and identified differentially methylated genes involved in ovarian activity. This study provides fundamental knowledge for elucidating the methylation mechanisms involved in ovarian activity, with a view to increasing hircine ovulation and kidding rates in the future.

## Methods

### Ovary collection and genomic DNA extraction

Guanzhong dairy goats, a well-defined native breed of Chinese goats, were used in this study. There were around 12 million dairy goats reared in China in 2012 [35], and Guanzhong dairy goats accounted for around 10% of them. The annual average milk yield of Guanzhong dairy goats is generally 500–600 kg, and their annual average litter size is 1.6–2.0 [36, 37]. Ten healthy, 3 years old pluriparous dairy goats were selected based on long-term observations and records of the oestrous cycles in each goat. Oestrus was taken as day zero, and five goats were slaughtered on day 10 of their oestrous cycle (ES samples). The other five goats were slaughtered at their next oestrus (DS samples). Ovary samples were collected and washed briefly with phosphate-buffered saline, then immediately frozen in liquid nitrogen until DNA extraction. Genomic DNA was extracted using a TIANamp Genomic DNA Kit (Tiangen, Beijing, China) according to the manufacturer’s instructions. RNase was used to lyse any potentially contaminating RNA and to ensure collection of pure DNA. The total quantity and purity of the DNA were analysed with a Bioanalyser 2100 (Agilent, CA, USA). OD_260/280_ ratios of 1.8–2.0 were considered to be good samples.

### Whole-genome bisulphite sequencing and data processing

Each genome library was constructed by pooling 5 μg of the homogenised total DNA from ES and DS ovary samples that came from different Guanzhong dairy goats. Genomic DNAs were fragmented into 100–300 bp sections by sonication (Covaris, Beijing, China) and purified with a MiniElute PCR Purification Kit (QIAGEN, Redwood, USA). The fragmented DNAs were end-repaired, and a single ‘A’ nucleotide was added to the 3′ end of the blunt fragments. The genomic fragments were ligated into methylated sequencing adapters. Fragments with the adapters were converted with bisulphite using a Methylation-Gold kit (ZYMO, Los Angeles, USA). The converted DNA fragments were PCR amplified [38] and sequenced using an Illumina HiSeq 2500 by Gene Denovo Biotechnology Co. (Guangzhou, China). Two lanes were used for sequencing the libraries (about 100G/line). Raw reads were filtered according to the following rules to obtain high-quality clean reads: 1) remove reads containing more than 10% of unknown nucleotides (N), and 2) remove low-quality reads containing more than 40% low-quality bases (quality score ≤ 20).

### Methylation level analysis

The obtained clean reads were mapped to the *Capra aegagrus hircus* reference genome using BSMAP software [39] (version: 2.90). A custom Perl script was used to call methylated cytosine and calculate the methylation level, based on the percentage of methylated cytosine in the entire genome, in each chromosome, and in different regions of the genome, for each type of sequence (CpG, CHG, and CHH) [40]. The methylation profile at flanking 2 kb regions and gene bodies (or transposable elements) was plotted based on the average methylation levels for each 100 bp interval, to assess methylation patterns in different genomic regions [40, 41].

### Analysis of differentially methylated regions

Differentially methylated regions in the two samples for each type of target (CpG, CHG, and CHH) were identified according to the following stringent criteria: a) The differentially methylated regions analyzed in the two groups of samples should include at least five cytosine sites that are methylated in at least one sample; b) The total depth of sequencing for each methylation cytosine site is > 10, and the depth of support for methylation cytosine is > 4; c) The region length is between 40 bp and 10 kb; d) The distance between adjacent methylated sites is < 200 bp; e) The fold change of the average methylation level > 2; and f) The Pearson’s chi-square test (χ^2^) value *P* ≤ 0.05. The putative differentially methylated regions overlapping at adjacent 2 kb (upstream or downstream) of the body regions of genes or transposable elements (TEs) were sorted for further study.

### Enrichment analysis of differentially methylated genes

Gene Ontology (GO) enrichment analysis was conducted to determine which terms were significantly enriched in differentially methylated genes compared with the genome background. Differentially methylated genes that corresponded to biological functions were filtered. All differentially methylated genes were mapped to GO terms in the Gene Ontology database (http://www.geneontology.org/). Gene numbers were calculated for every term. Significantly enriched GO terms in differentially methylated genes in comparison with the genome background were defined by the hypergeometric test. *P*-values [42, 43] were calculated by

$$ P=1-\sum \limits_{i=0}^{m-1}\frac{\left(\begin{array}{c}M\\ {}i\end{array}\right)\left(\begin{array}{c}N-M\\ {}n-i\end{array}\right)}{\left(\begin{array}{c}N\\ {}n\end{array}\right)}, $$

where *N* is the number of all genes with GO annotation; *n* is the number of differentially methylated genes in *N*; *M* is the number of all genes annotated to certain GO terms; and *m* is the number of differentially methylated genes in *M*. The calculated *P*-values were subjected to Bonferroni correction, with Q-values ≤0.05 as a threshold. GO terms that satisfied this condition were defined as significantly enriched in differentially methylated genes. This analysis was performed to recognise the main biological functions of differentially methylated genes.

Genes that usually interact with one another and participate in certain biological functions were determined. Pathway-based analysis helps to understand the biological functions of genes. KEGG is a major, publicly available pathway-related database [44]. Pathway enrichment analysis was conducted to identify significantly enriched metabolic pathways or signal transduction pathways in differentially methylated genes compared with the entire genome background. The calculation formula was similar to that used for GO analysis, with minor modifications. Here, *N* is the number of all genes with KEGG annotation, *n* is the number of differentially methylated genes in *N*, *M* is the number of all genes annotated to specific pathways and *m* is the number of differentially methylated genes in *M*. The calculated *P*-values were subjected to Bonferroni correction, with Q-values ≤0.05 as a threshold. Pathways satisfying this condition were defined as significantly enriched in differentially methylated genes.

## Results

### Summary of methylome sequencing

Table 1 shows a statistical summary of the sequencing parameters. A total of 643,846,892 (ES) and 678,107,258 (DS) unique clean reads were obtained from whole-genome bisulphite sequencing (WGBS). The mapped ratios were 86.34% in the ES group and 88.05% in the DS group, with corresponding sequence depths of 33.03 and 35.47, respectively. The results identified methylated regions, covering the entire genome, with sufficient depth and at high resolution.

**Table 1:** Summary of sequencing results

Samples	Raw reads	Clean reads	Clean data	Q20	GC	Mapped reads	Mapped ratio	Sequence depth
ES	651,655,384	643,846,892	96,385,663,457 bp	93.07%	21.08%	555,925,818	86.34%	33.03
DS	681,967,800	678,107,258	101,611,244,110 bp	96.03%	20.47%	597,077,755	88.05%	35.47

**Fig. 1:**
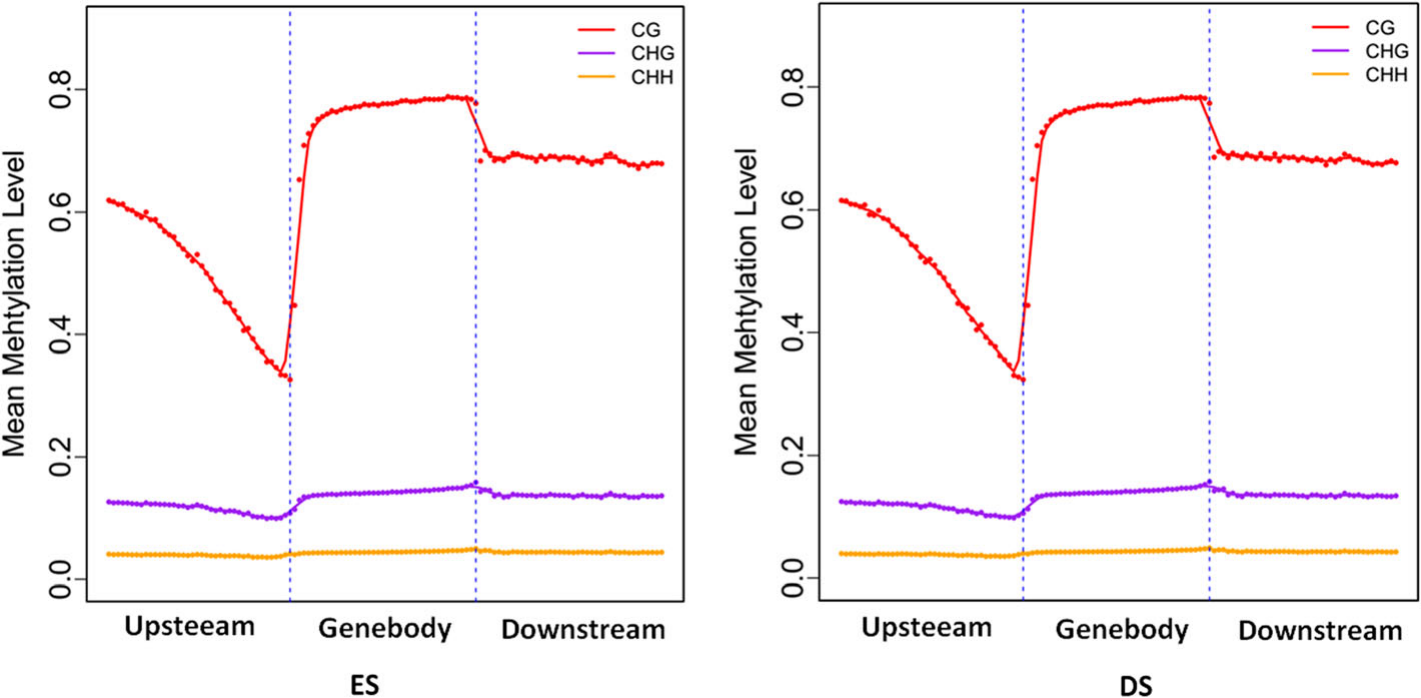
Methylation patterns of hircine ovarian genomic features. Gene regions are characterised as: 2 kb upstream of the CDS start, gene body, and 2 kb downstream of the CDS end. Each dot denotes the mean methylation level per bin and the respective lines denote the 5-bin moving average. The y-axis is the average methylation level for each dot. H = A/T/C

**Fig. 2:**
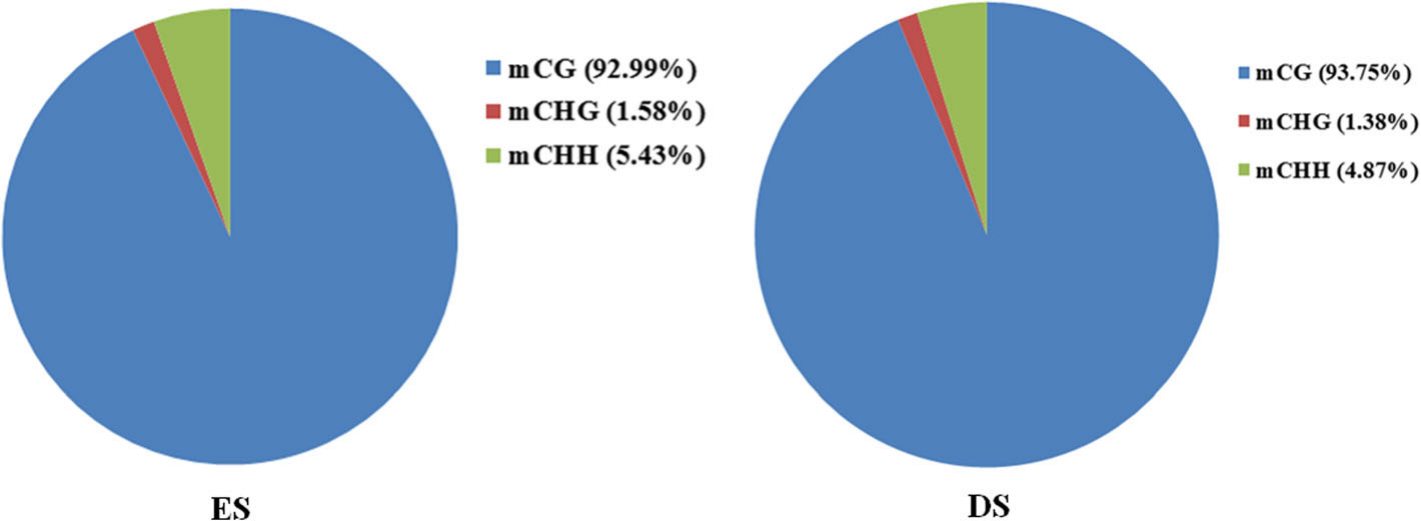
Distribution of the different types of methylated C bases. The pie indicates the percentage of methylated C in total mC. The horizontal axis of the graph indicates the methylation level with each 10% being the first gear, and the vertical axis indicating the specific methylation level of mC in all mCs in C

Additional file 1: Table S1 shows the distribution of WGBS reads in different chromosomes (chromosomes 1–29 and chromosome X). The distribution of WGBS reads in different genome regions represents a genome-wide methylation pattern. To understand DNA methylation levels of various functional genomic elements, we classified the distribution of the WGBS reads into: upstream region (2,000 bp), gene body region, downstream region (2,000 bp), exon, intron, CDS and 5′- and 3′-UTR regions (Additional file 2: Table S2). The highest DNA methylation levels were found in introns and exons, followed by 3′- and 5′-UTR regions, while the lowest level was detected in the upstream region. In the promoter regions, the proximal regions (− 200 bp to + 500 bp) exhibited the lowest DNA methylation levels (Fig. 1). Furthermore, cytosine methylation exclusively comprised CpG methylation, but occasionally methylation was found in CHH and CHG (Fig. 2).

### Characterisation of differentially methylated regions-related genes

Compared with the DS group, 26,910 differentially methylated regions had a higher methylation level and 21,453 differentially methylated regions had a lower methylation level in the ES group (Additional file 3: Table S3). Differentially methylated regions were mapped to each chromosome and annotated to find differentially methylated genes. Three differentially methylated genes were found in the gene body region in CHH content methylation, and 32 differentially methylated genes in CHG content methylation. In addition, one differentially methylated gene was found in the upstream region in CHG content methylation (Additional file 4: Table S4). In CG content methylation, the most methylated parts were gene body regions, with 3,446 differentially methylated genes, but it was lower in upstream regions, with 647 differentially methylated genes (Additional file 4: Table S4). Compared with the DS samples, the methylation ratios of *STAR*, *FGF2*, *FGF12*, *BMP5* and *SMAD6* genes were lower in the ES samples (*P*-values ≤0.05 and fold change of methylation ratios ≥2). The gene methylation ratios of *EGFR*, *TGFBR2*, *IGF2BP1* and *MMD2* were higher in the ES samples (Additional file 4: Table S4).

### GO annotation and KEGG pathways of differentially methylated genes

Regulatory networks of differentially methylated genes were constructed to reveal differentially methylated gene-regulated GOs and signalling pathways, and to understand the physiological functions and biological processes associated with differentially methylated genes in ovarian activity. In this study, GO enrichments of the differentially methylated genes were categorised into 124 functional groups (Q-value ≤0.05). The numbers of terms enriched were: 33 in molecular functions, 19 in cellular components, and 72 in biological processes (Additional file 5: Table S5). Most GO enrichments occurred in CpG islands with 118 functional groups (Additional file 5: Table S5). No GO enrichments were found in the CHH content sequence. In cellular components, the most significantly enriched GO terms included cell projection (GO:0042995), with 114 differentially methylated genes, followed by cell periphery (GO:0071944), endomembrane system (GO:0012505) and plasma membrane (GO:0005886). The most significant functional terms in biological processes were multicellular organismal process (GO:0032501), with 438 differentially methylated genes. For molecular functions, the most significant functional terms were those that bound to 958 differentially methylated genes (GO:0005488) (Additional file 5: Table S5). KEGG enrichment analysis of differentially methylated gene-related genes was also conducted for hypermethylated and hypomethylated genes. Overall, the differentially methylated genes were significantly enriched in 80 KEGG pathways, with Q-values ≤0.05. The KEGG pathways with the most genes related to differentially methylated genes included the pathways in cancer (KO05200), with 121 genes, followed by the PI3K-Akt signalling pathway (KO04151), with 92 genes, and focal adhesion (KO04510), with 82 genes. In addition, 223 differentially methylated genes were found in the gonadotropin-releasing hormone (GnRH) signalling pathway (KO04912), ovarian steroidogenesis pathway (KO04913), oestrogen signalling pathway (KO04915), oxytocin signalling pathway (KO04921), insulin secretion pathway (KO04911) and mitogen-activated protein kinase (MAPK) signalling pathway (KO04010) (Additional file 6: Table S6). The GnRH (KO04912) and oestrogen signalling pathways (KO04915) were the most significantly enriched pathways in hypermethylated genes (Additional file 7: Table S7).

## Discussion

The domestic goat, *Capra aegagrus hircus,* is an important, globally distributed livestock species, particularly in developing countries. The global goat population reached more than 1 billion animals in 2017. The main capacity constraint for goats is their litter size, which is dependent on a healthy reproductive cycle. DNA methylation regulates sexual and ovarian maturation [24, 25]. By profiling the methylome of goat ovaries, the present study demonstrated methylation changes during the oestrous cycle. The sequence depths were 33.03 and 35.47 in the oestrus (ES) and dioestrus (DS) groups, respectively. The mapped ratios in the two groups were relatively high, and the WGBS results were satisfactory for the analysis. The methylation patterns of hircine genes are similar to those of other vertebrates, such as humans [45], sheep [46], cows [19] and horses [47]. The methylation level in gene bodies was higher than in the upstream and downstream regions. The methylation level between upstream regions and gene bodies increased sharply but then decreased after the gene bodies. Hence, exons exhibited higher methylation levels than introns; this finding is consistent with patterns seen in humans [48] but differs from those seen in porcine ovaries [49]. The difference in the results may be due to differences in sequencing methods or species specificity. The present study used WGBS in hircine ovaries, whereas the previous study used reduced representation bisulphite sequencing in porcine ovaries. Further analysis in our study demonstrated that the coding sequence (CDS) had the most enriched methylation cytosine in the genome.

The ovaries undergo dynamic morphological changes during the oestrous cycle. Ovarian follicles grow continuously and secrete hormones during dioestrus, and the dominant follicles release oocytes to start luteinisation during oestrus [50]. These phenomena depend on a series of factors, such as gonadal hormones and growth factors [29]. However, the effect of DNA methylation patterns of genes on ovarian changes during the oestrous cycle remains unclear. A total of 48,363 differentially methylated regions were found between the DS and ES groups, and these differentially methylated regions belonged to 4,567 genes. Many genes related to ovarian activity showed low methylation levels, including *FGF2*, *BMP5*, *NR5A2* and *STAR*. Two of them, *FGF2* and *BMP5*, are necessary for cell proliferation, *NR5A2* is essential for corpus luteum generation [51–54] and *STAR* encodes a key protein that participates in hormone synthesis [55]. Low methylation levels can facilitate gene transcription, and genes with low methylation levels may also help with corpus luteum generation and progesterone secretion after oestrous. *FGF9* and *Bcl2*, antagonists of cell death, had high methylation levels in the ES group. *FGF9* is highly expressed in large follicles, and contributes to follicle development and steroidogenesis in dairy cattle [56]. The high methylation level of the *FGF* gene can be attributed to follicle luteinisation, which prevents the growth of small follicles during oestrous. Previous studies and our investigations showed that the *NR5A2*, *STAR*, *FGF2* and *BMP5* genes might have potential application value in regulating caprine oestrus. Furthermore, we identified cell-type makers and potential lineage markers in follicular development that showed different methylation levels; these markers included cadherin-12 (*CDH12*), regulators of G-protein signalling (*RGSs*) and NIPA-like protein 3 (*NIPAL3*) [57]. In addition, several regions (chromosomes 5 and 17) associated with the same gene or gene cluster (*DGCR6L*, *HOXC4*, *HOXC5*, *HOXC6*, *HOXC8*, *HOXC9* and *HOXC10*) can be differentially methylated (Additional file 4: Table S4). The loci identified may display a DNA methylation distribution consistent with uniparental effects, where one of the alleles led to a larger average phenotypic value than the other [58, 59].

Gene Ontology annotation provides a controlled vocabulary to comprehensively describe the properties of genes and gene products. In this study, differentially methylated genes were enriched in 168 functional groups. The results indicated that the functional groups of genes have different methylation levels between oestrus and dioestrus, providing an overview of dynamic changes in ovarian function. Pathway analysis revealed the involvement of methylated genes in the two groups in temporal ovarian signalling. The ovaries undergo cycles of proliferation, differentiation, ovulation and corpus luteum generation [60]. These processes depend on a series of signalling pathways. In this study, some differentially methylated genes were enriched in the gonadotropin-releasing hormone (GnRH) signalling pathway. GnRH is a key regulator in the hypothalamic-pituitary-gonadal axis. The GnRH pathway alters follicle growth, secretion and ovulation [29, 61]. The ovaries also undergo various biological processes during the oestrous cycle. Follicle growth and hormone secretion occur in the dioestrous phase, whereas ovulation occurs in the oestrous phase [22]. A total of 223 differentially methylated genes were enriched in the GnRH pathway, thereby confirming that this pathway is essential for ovarian activity. Furthermore, other enriched pathways, such as ovarian steroidogenesis pathway (KO04913), the oestrogen signalling pathway (KO04915) and the PI3K-Akt signalling pathway, are crucial for ovarian activity [62, 63]. The present study provides valuable information for future research into the molecular mechanisms underlying the progression of ovarian activity in goats.

## Conclusions

This study is the first large-scale comparison of the high-resolution DNA methylation landscapes of oestrous and dioestrous ovaries from dairy goats. Previous studies and our investigations have shown that the *NR5A2*, *STAR*, *FGF2* and *BMP5* genes might have potential application value for regulating caprine oestrus.

## Additional files

Additional file 1: **Table S1.** Distribution of WGBS reads in different chromosomes. (XLSX 18 kb)

Additional file 2: **Table S2.** Distribution of three mC types in gene regions. (XLSX 11 kb)

Additional file 3: **Table S3.** Three types (CpG, CHG and CHH) of methylation characterisation of differentially methylated regions. The fold change of the average methylation level > 2 and Pearson’s chi-square test (χ^2^) value *P* ≤ 0.05. H = A/T/C. (XLSX 4649 kb)

Additional file 4: **Table S4.** Differentially methylated genes of the three types of methylated C bases. (XLSX 1582 kb)

Additional file 5: **Table S5.** Functional categorisation of methylated CpG related genes. (XLSX 48 kb)

Additional file 6: **Table S6.** KEGG pathways of differentially methylated genes in DS versus ES. (XLSX 37 kb)

Additional file 7: **Table S7.** KEGG pathway of hypermethylated and hypomethylated genes in DS versus ES. (XLSX 38 kb)

## Abbreviations

*BMP5*: Bone morphogenetic protein 5
DS: Dioestrous phase
*EGFR*: Epidermal growth factor receptor
ES: Oestrous phase
*FGF2*, *12*: Fibroblast growth factors 2 and 12
*IGF2BP1*: Insulin-like growth factor 2 mRNA-binding protein 1
*MMD2*: Monocyte-to-macrophage differentiation factor 2
*SMAD6*: SMAD family member 6
*STAR*: Steroidogenic acute regulatory
TEs: Transposable elements
*TGFBR2*: TGF-beta receptor type 2
